# Generalized Optical Design of the Double-Row Circular Multi-Pass Cell

**DOI:** 10.3390/s18082680

**Published:** 2018-08-15

**Authors:** Zheng Yang, Yin Guo, Xianshun Ming, Liqun Sun

**Affiliations:** State Key Laboratory of Precision Measurement Technology and Instruments, Department of Precision Instruments, Tsinghua University, Beijing 100084, China; yang-z14@mails.tsinghua.edu.cn (Z.Y.); y-guo13@mails.tsinghua.edu.cn (Y.G.); mxs14@mails.tsinghua.edu.cn (X.M.)

**Keywords:** gas sensing, absorption spectroscopy, multi-pass cell, geometric optical design

## Abstract

A new design of circular multi-pass cells (CMPC) with two rows of reflection spots on mirrors is presented. The effective optical path length (OPL) of this novel CMPC is double that of traditional CMPC with the same diameter and interval of spots. This OPL can be readily adjusted to have regular intervals by rough rotation adjustment. We achieved a spatial separation of pre- and post-transfer optical systems that was adequately large even in the cases with a large number of passes. Analytical chief ray tracing analysis and a generalized method for parameter determination for designing the cell are presented in detail. The stable condition of the double-row CMPC (DR-CMPC) is also derived by the ABCD matrix method. Designs with maximum effective OPL of 74.72 m, 48.67 m and 24.57 m are demonstrated and verified by ray tracing simulations within a 25 cm diameter DR-CMPC. An adjustment of the regular intervals to 1 m can be achieved in both designs. The overall astigmatism of the design with an effective OPL of 74.72 m is only 9.30 × 10^−6^ mm, which is four orders of magnitude smaller than that of the traditional CMPC with similar geometric parameters.

## 1. Introduction

Multi-pass cells (MPCs) with a long effective optical path length (OPL) in a compact volume are of increasing importance in laser absorption spectroscopy for trace gas sensing, as these properties enhance the absorption spectroscopy signals of gaseous samples with extremely low concentrations or weak absorption line strengths, in addition to reducing the instrumental footprint. Various MPCs have been developed for this purpose, such as the White cell [1] and the Herriott cell [2], which have the simplest configurations and can be easily implemented. However, the effective areas where the laser beams can be reflected on the mirrors in these two basic designs are relatively small. The original White-type cell [1] has only one row of reflection spots on the field mirror, while the early phase of improvements that were proposed by Berstein et al. [3] and Pickett et al. [4] have only two rows, which also possesses a relatively low path-to-volume ratio (PVR). Several latter variations aimed to promote the PVR, such as the multi-pass matrix systems (MMS) proposed by Chernin et al. [5,6,7], Glowacki et al. [8] and Guo [9]. Just as its name implies, the reflection spots are arranged to form a matrix on the field mirrors of MMS and the interval of rows and columns can be easily adjusted. Consequently, a large PVR and readily adjustable effective OPL are achieved in MMS. However, such configurations usually need a relatively longer basic length (about 0.2–1 m or even longer), which makes the system cumbersome as it can have a volume of up to five liters [10]. The Herriott cell [2] shares a similar drawback in PVR as the original White-type cell, because the reflection spots on the mirrors only emerge in an elliptical pattern within the Herriott-type cell. The astigmatic Herriott-type cell is an important improvement of the Herriott cell, which was presented and developed by Herriott et al. [11] and McManus et al. [12]. The key innovation point of this astigmatic Heriott-type cell involves its use of astigmatic mirrors, which possess different radii of curvatures in the directions of the X-axis and Y-axis in order to replace the spherical mirrors in the Herriott cell. Consequently, the reflection spots form Lissajous patterns on the mirrors and result in an improved PVR. However, the extremely strict accuracy requirements for manufacture and adjustment of the mirrors limits the applications of the astigmatic Herriott-type cell. Although a method of rotating one mirror about its optical axis was presented for compensating the manufacture errors on the radii of astigmatic mirrors [12], this results in additional complexity in the alignment process.

The idea of circular MPCs (CMPCs) was first proposed by Chernin et al. [13,14] and a similar design was adopted by Ofner et al. [15], in which the laser beam enters into a single circular mirror with a high-reflectivity (HR) coated internal surface at a certain incident angle. After this, the beam reflects back and forth, before forming a portion of a regular star polygon pattern in the same plane on the side wall. It was partially different from the circular cells that have been commonly used in the past few years, with the most crucial difference involving the incident and exit holes being separated on the side wall in this primordial circular cell (that is why the reflection spot patterns are only a portion of, rather than an intact, regular star polygon pattern). In more recent designs, the laser beams enter and exit the cell through the same hole. The key advantage of a separated entrance port and exit port relies on the avoidance of overlapping of the pre- and post-transfer optical systems, but it limits the adjustable range of the incident angle and thus the range of effective OPL that can be achieved within the cells. Riedel et al. [16], Bernacki [17] and Tuzson et al. [10,18,19,20,21] proposed and developed CMPCs in which the entrance and exit beams share the same hole on the side wall, which holds the advantages of compact size, easy alignment and adjustable OPL in different detection manners. However, the reflection spots only appear circularly in the same plane on the internal wall (with the beam reflection trajectory forming an entire regular star polygon pattern) in the CMPC. This means that if one wants to increase the effective OPL of the CMPC, the only way is to decrease the incident angle of the beam, *θ*. However, this will lead to a reduction in the interval of adjacent spots and hence the interference fringes become severe. Although the off-axis beam pattern that was proposed by Mangold et al. [20] helps to alleviate the problem by dividing the spots into two planes on the side wall, the spots are not homogeneously distributed on each plane, as some of the spots keep the same interval as the cases without the off-axis pattern. Thus, the corresponding components of interference fringes still exist. Another disadvantage caused by a decrease in the incident angle is that the pre- and post-transfer optical systems get too close in space, which is inconvenient for the optical component arrangement in both systems. Furthermore, the adjustment of effective OPL within the traditional CMPC can only be achieved by changing the incident angle, which demands extremely high precision of angular adjustment, especially in the cases with a large number of reflections. For example, the incident angle *θ* = 2.400° corresponds to 150 passes within the CMPC (the corresponding pattern is a regular star polygon with parameters *p* = 150 and *q* = 73), while *θ* = 2.384° corresponds to 302 passes. This means that a misalignment of only 0.016° in the incident angle adjustment can make a difference of 152 passes within the cell. Therefore, we need a better method to prevent the need to change the incident angle frequently to achieve a convenient adjustment of the effective OPL.

In this work, a new version of a circular multi-pass cell is proposed for the first time, which consists of two identical high-reflectance coated mirrors with spherical inner surfaces. The two mirrors are piled up vertically and share the same axis of rotational symmetry. Two regular star polygon patterns (both patterns are the same as the patterns in traditional CMPC) are formed simultaneously on different horizontal planes that lie on the two mirrors, respectively. Therefore, we can achieve a longer effective OPL that is double that of the traditional circular cell without increasing the interference fringes, which only requires a slight modification to the incident angle as discussed in Section 2. The entrance and exit holes are spatially separated to avoid overlap of the pre- and post-transfer optics. An equally spaced adjustment of effective OPL can be easily implemented by rotating one of the mirrors around the axis of rotational symmetry with a relatively rough precision of only a few degrees without any change in incident angle and pattern of spots. The stability of the DR-CMPC is analyzed by the ABCD matrix method. The methods for selecting the initial parameters and determining the derived parameters within the double-row CMPCs (DR-CMPCs) are also proposed. Furthermore, three simulation examples with a maximum effective OPL of 74.3 m, 48.67 m and 24.57 m are demonstrated in Section 4, which all have an adjustment to a regular interval of 1 m. The overall astigmatism of the design with an effective OPL of 74.72 m is only 9.30 × 10^−6^ mm, which is four orders of magnitude smaller than that of the traditional CMPC with similar geometric parameters.

## 2. Analytical Tracing of Circular Multi-Pass Cell

### 2.1. Traditional Circular Multi-Pass Cell

**Figure 1:**
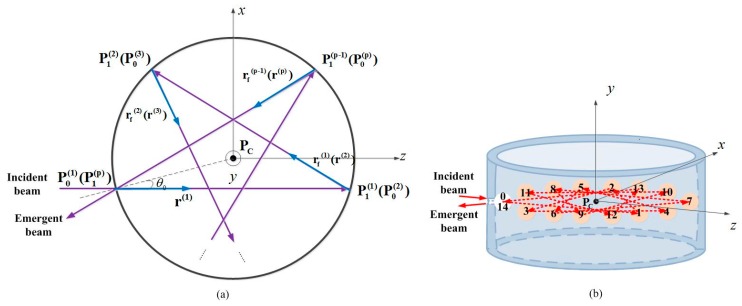
(**a**) Top view of the successive reflections on the spherical mirrors within the traditional circular multi-pass cell (CMPC); $\theta_{0}$ is the incident angle of the beam, $P_{C}=(0,0,0)$ is the common center of curvature of each spherical reflection within the cell, the initial direction vector of the incident beam is $r^{\left(1\right)}=(0,0,1)$ and the initial incident point is $P_{0}^{\left(1\right)}=(-R\sin\theta_{0},0,-R\cos\theta_{0})$; *z*^(*i*)^ is the optical path length (superscript *i* denotes the *i*th reflection); $r^{\left(i\right)}$,$r_{f}^{\left(i\right)}$ and $r_{N}^{\left(i\right)}$ are the direction vectors of the incident beam and the reflective beam as well as the normal of the reflection plane, respectively; $P_{0}^{\left(i\right)}$, $P_{1}^{\left(i\right)}$ and $P_{C}^{\left(i\right)}$ are the coordinate values of the incident point and the reflective point as well as the center of curvature, respectively; and *R* is the radius of curvature of the internal spherical surface; (**b**) A 14-passes example (with number of passes *p* = 14 and beam density of the *p*-sided star polygon pattern *q* = 5) in three-dimensional view (the height of the circular mirror is exaggerated for clarity). The solid/dotted arrow shows the propagation path of the beam outside/inside the CMPC; pink circles represent the positions where the beams bounce off the internal surface, while the numbers on the circles represent the order in which the beam arrives; the white circle with 0 (for incident) and 14 (for exit) represents the position of incident/exit hole.

The spherical equations are useful for chief ray tracing of sequential reflections in MPCs, especially in CMPCs, because the results can verify or guide the parametric design of the cell. The top view of the successive reflections on the spherical mirrors within the CMPCs is illustrated in Figure 1a, which can be described by the following spherical equations:

(1) $$z^{\left(i\right)}=-r^{\left(i\right)}•(P_{0}^{\left(i\right)}-P_{C}^{\left(i\right)})+\sqrt{{[r^{\left(i\right)}•(P_{0}^{\left(i\right)}-P_{C}^{\left(i\right)})]}^{2}-(P_{0}^{\left(i\right)}-P_{C}^{\left(i\right)})•(P_{0}^{\left(i\right)}-P_{C}^{\left(i\right)})+R^{2}},$$

(2) $$P_{1}^{\left(i\right)}=P_{0}^{\left(i\right)}+z^{\left(i\right)}r^{\left(i\right)},$$

(3) $$r_{N}^{\left(i\right)}=(P_{1}^{\left(i\right)}-P_{C}^{\left(i\right)})/\sqrt{(P_{1}^{\left(i\right)}-P_{C}^{\left(i\right)})•(P_{1}^{\left(i\right)}-P_{C}^{\left(i\right)})}=(P_{1}^{\left(i\right)}-P_{C}^{\left(i\right)})/R,$$

(4) $$r_{f}^{\left(i\right)}=r^{\left(i\right)}-(2r^{\left(i\right)}•r_{N}^{\left(i\right)})r_{N}^{\left(i\right)},$$

where $z^{\left(i\right)}$ is the optical path length (superscript *i* denotes the *i*th reflection); $r^{\left(i\right)}$, $r_{f}^{\left(i\right)}$ and $r_{N}^{\left(i\right)}$ are the direction vectors of the incident beam and the reflective beam as well as the normal of the reflection plane, respectively; $P_{0}^{\left(i\right)}$, $P_{1}^{\left(i\right)}$ and $P_{C}^{\left(i\right)}$ are the coordinate values of the incident point and the reflective point as well as the center of curvature, respectively; and *R* is the radius of curvature of the internal spherical surface. Because each reflective point and direction vector of the reflective beam can be considered as the incident point and direction vector of the incident beam of the next reflection, a recursion formula can be expressed as:

(5) $$P_{0}^{\left(i+1\right)}=P_{1}^{\left(i\right)},$$

(6) $$r^{\left(i+1\right)}=r_{f}^{\left(i\right)},$$

For arbitrary reflections on the internal spherical surface of the circular mirror, the centers of curvature are always $P_{C}^{\left(i\right)}\equiv (0,0,0)$. We can obtain the reflective point and direction vector of the reflective beam of the first reflection via Equations (1)–(4):

(7) $$P_{1}^{\left(1\right)}=(-R\sin\theta_{0},0,R\cos\theta_{0}),$$

(8) $$r_{f}^{\left(1\right)}=(\sin2\theta_{0},0,-\cos2\theta_{0}).$$

According to Equations (5) and (6), the direction vector and incident point of the second reflection are $r^{\left(2\right)}=(\sin2\theta_{0},0,-\cos2\theta_{0})$ and $P_{0}^{\left(2\right)}=(-R\sin\theta_{0},0,-R\cos\theta_{0})$, respectively. By iteratively using Equations (1)–(6) in this way, the ray tracing of the following reflections can be achieved. By mathematical induction, the general expressions of the reflective point of the *n*th reflection within traditional CMPC are attained as:

(9) $$P_{1}^{\left(n\right)}=({\left(-1\right)}^{n}R\sin(2n-1)\theta_{0},0,{\left(-1\right)}^{n-1}R\cos(2n-1)\theta_{0}).$$

To validate the general expressions, we utilized Equation (9) to verify the result of the relationship between the number of passes *p* and incident angle $\theta_{0}$. This has been discussed in a previous publication by the polygon geometry method [10]:

(10) $$\theta_{0}=\frac{p-2q}{2p}\pi ,$$

where *p* indicates the total number of sides of the regular star polygon pattern, and *q* indicates that, on the circumscribed circle of the regular star polygon pattern, the corresponding arc of one side is divided into *q* segments by other vertexes. Besides, *p* and *q* are relative prime integers. When Equation (10) is fulfilled, the beam will go through *p* passes within the cell and exit at the same position as it enters. This means that $P_{0}^{\left(1\right)}=P_{1}^{\left(p\right)}$ in this case. We substituted $\theta_{0}$ of Equation (10) into Equation (9), which provides the following:

(11) $$\begin{array}{ll} P_{1}^{\left(p\right)} & =({\left(-1\right)}^{p}R\sin(2p-1)\theta_{0},0,{\left(-1\right)}^{p-1}R\cos(2p-1)\theta_{0})\\ & =({\left(-1\right)}^{p}R\underset{\sin(n\pi -\theta_{0})={\left(-1\right)}^{n+1}\sin\theta_{0}}{\underset{︸}{\sin[(p-2q)\pi -\theta_{0}]}},0,{\left(-1\right)}^{p-1}R\underset{\cos(n\pi -\theta_{0})={\left(-1\right)}^{n}\cos\theta_{0}}{\underset{︸}{\cos[(p-2q)\pi -\theta_{0}]}})\\ & =({\left(-1\right)}^{2(p-q)+1}R\sin\theta_{0},0,{\left(-1\right)}^{2(p-q)-1}R\cos\theta_{0})\\ & =(-R\sin\theta_{0},0,-R\cos\theta_{0})=P_{0}^{\left(1\right)} \end{array}.$$

Therefore, the previous polygon geometry results in Equation (10) agree with the analytical ray tracing results obtained using spherical equations. Therefore, the validity of our method can be guaranteed. A 14-passes example (*p* = 14, *q* = 5) of the traditional CMPC is illustrated in Figure 1b.

### 2.2. Double-Row Circular Multi-Pass Cell

**Figure 2:**
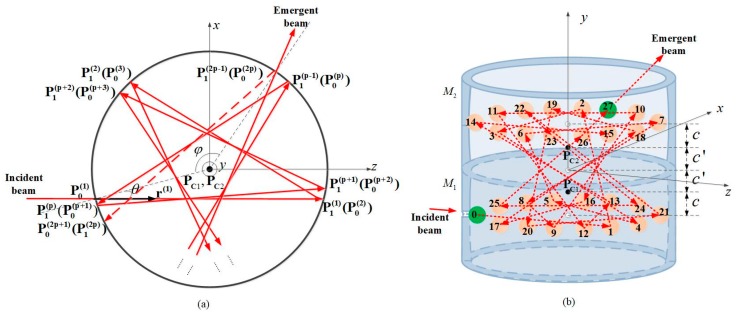
(**a**) The top view of the successive reflections on the spherical mirrors within the double-row circular multi-pass cell (DR-CMPC), is the incident angle of the beam; is the angle between the incident and exit holes projected into x-O-z plane; $r^{\left(i\right)}$, $r_{N}^{\left(i\right)}$ and $r_{f}^{\left(i\right)}$ are the direction vectors of the incident beam, normal and reflective beam, respectively, and are the coordinate values of the incident point and reflective point, respectively, $P_{0}^{\left(i\right)}$ and $P_{1}^{\left(i\right)}$ are the centers of curvature of the circular mirrors, $M_{1}$ and $M_{2}$, respectively; (**b**) A 27-passes example (*p* = 14, *q* = 5) in three-dimensional view (the height of the circular mirror is exaggerated for clarity). The solid/dotted arrow shows the propagation path of the beam outside/inside the DR-CMPC; pink circles represent the positions where the beam bounces off the internal surface, while the numbers on the circles represent the order in which the beam arrives; green circles with 0 (for incident) and 27 (for exit) represent the position of incident and exit holes, respectively. Compared to Figure 1b, the number of passes has almost doubled, while the parameters of *p* and *q* remain the same (namely the density of spots and thus, the interference fringes are not increased).

DR-CMPC consists of two identical circular mirrors with offset spherical internal surfaces. In contrast to the traditional case, the spherical centers of the offset spherical internal surfaces are not in the planes that the reflection spots lie on, which is shown in Figure 2b. Therefore, the reflection beam within the cell transitions back and forth on the two offset circular mirrors, which emerges with approximately double of the same sets of reflection spots as in the original design (thus double the effective OPL) in two planes. After this, in the top view, two regular star polygon patterns (slightly non-coincidental, which will be discussed below) appear within the cell as shown in Figure 2a.

A similar discussion according to the spherical equations can be used to deduce the chief ray tracing within the DR-CMPC. The initial direction vector of the incident beam is $r^{\left(1\right)}=(0,0,1)$ and the initial incident point is $P_{0}^{\left(1\right)}=(-\sqrt{R^{2}-c^{2}}\sin\theta ,c+c^{\prime },-\sqrt{R^{2}-c^{2}}\cos\theta )$, where *c* is the distance between the center of curvature and the plane that the reflection spots lie on and *c’* is the distance between the center of curvature and the interface of the two circular mirrors, which was illustrated in Figure 2b. In contrast to the traditional design, the center of the curvatures change for different reflections in a cycle of four, which is written as:

(12) $$P_{C}^{\left(i\right)}=\{\begin{array}{l} P_{C1}=(0,-c^{\prime },0)(i=4k+1,k=0,1,2\cdots )\\ P_{C2}=(0,c^{\prime },0)(i=4k+2,k=0,1,2\cdots )\\ P_{C2}=(0,c^{\prime },0)(i=4k+3,k=0,1,2\cdots )\\ P_{C1}=(0,-c^{\prime },0)(i=4k+4,k=0,1,2\cdots ) \end{array}.$$

For ease of calculation, we tentatively set:

(13) $$c^{\prime }=c\cos\theta .$$

After this, the reflective point and direction vector of the reflective beam of the first reflection within the DR-CMPC can be obtained by Equations (1)–(4) as:

(14) $$P_{1}^{\left(1\right)}=(-\sqrt{R^{2}-c^{2}}\sin\theta ,-2c\cos^{2}\theta ,\sqrt{R^{2}-c^{2}}\cos\theta ),$$

(15) $$r_{f}^{\left(1\right)}=((1-\frac{c^{2}}{R^{2}})\sin2\theta ,\frac{2c}{R}\sqrt{1-\frac{c^{2}}{R^{2}}}\cos\theta ,\frac{c^{2}}{R^{2}}-(1-\frac{c^{2}}{R^{2}})\cos2\theta ).$$

To simplify the calculations of the following reflections, an approximate method is utilized to transform the expressions of the direction vector in Equation (15) into a simpler form, which is proportional to a cosine function and is similar to Equation (9). The degradation in the precision of ray tracing is acceptable. Similar to our trial calculation, the second-order approximation of the *c*/*R* term is adequate, which means that the cubic and the higher order terms can be neglected in the calculation below. Thus, we may transform the expression of $r_{f}^{\left(1\right)}$ in Equation (15) into:

(16) $$\begin{array}{c} r_{f}^{\left(1\right)}=((1-\frac{2c^{2}}{R^{2}}\cos^{2}\theta )\sin2\theta +\frac{c^{2}}{R^{2}}\sin2\theta \cos2\theta ,\frac{2c}{R}\cos\theta ,\\ \frac{c^{2}}{R^{2}}\sin^{2}2\theta -(1-\frac{2c^{2}}{R^{2}}\cos^{2}\theta )\cos2\theta ) \end{array}.$$

We defined a small angle ε as:

(17) $$\sin\varepsilon =\frac{c^{2}}{R^{2}}\sin2\theta .$$

Thus, the expression of  can be simplified as:

(18) $$r_{f}^{\left(1\right)}=((1-\frac{2c^{2}}{R^{2}}\cos^{2}\theta )\sin(2\theta +\varepsilon ),\frac{2c}{R}\cos\theta ,-(1-\frac{2c^{2}}{R^{2}}\cos^{2}\theta )\cos(2\theta +\varepsilon )).$$

After this, the analogous derivation by mathematical induction is applied to the ray tracing within DR-CMPC to find the general expressions of the *n*th reflection in the cases of n=4k−3,4k−2,4k−1,4k(k=1,2,…), respectively:

(19) $$\begin{array}{c} P_{1}^{\left(4k-3\right)}=(-R(1-\frac{c^{2}}{2R^{2}})\sin[(8k-7)\theta +(4k-4)\varepsilon ],-2c\cos^{2}\theta ,\\ R(1-\frac{c^{2}}{2R^{2}})\cos[(8k-7)\theta +(4k-4)\varepsilon ]), \end{array}$$

(20) $$\begin{array}{c} P_{1}^{\left(4k-2\right)}=(R(1-\frac{c^{2}}{2R^{2}})\sin[(8k-5)\theta +(4k-2)\varepsilon ],2c\cos^{2}\theta ,\\ -R(1-\frac{c^{2}}{2R^{2}})\cos[(8k-5)\theta +(4k-2)\varepsilon ]), \end{array}$$

(21) $$\begin{array}{c} P_{1}^{\left(4k-1\right)}=(-R(1-\frac{c^{2}}{2R^{2}})\sin[(8k-3)\theta +(4k-2)\varepsilon ],2c\cos^{2}\theta ,\\ R(1-\frac{c^{2}}{2R^{2}})\cos[(8k-3)\theta +(4k-2)\varepsilon ]), \end{array}$$

(22) $$P_{1}^{\left(4k\right)}=(R(1-\frac{c^{2}}{2R^{2}})\sin[(8k-1)\theta +4k\varepsilon ],-2c\cos^{2}\theta ,-R(1-\frac{c^{2}}{2R^{2}})\cos[(8k-1)\theta +4k\varepsilon ]).$$

By further calculation, we find that in the second-order approximation, the results of x- and z-coordinate values in Equations (19)–(22) can be generalized to cases with $c^{\prime }=c\cos\theta +\delta$, where $\delta <c^{2}/R$. We may set $\delta =2c\sin^{2}\theta$ and the incident angle *θ* meets the condition of:

(23) $$\sin\theta \leq \sqrt{\frac{c}{2R}}.$$

After this, the value of *c’* is equal to *c* and is not dependent on the incident angle *θ* in Equation (13). In this case, the general expressions of each reflection point of the chief ray are:

(24) $$\begin{array}{c} P_{1}^{\left(4k-3\right)}=(-R(1-\frac{c^{2}}{2R^{2}})\sin[(8k-7)\theta +(4k-4)\varepsilon ],\\ -2c+4c\sin\theta \frac{\cos[(4k-3)\theta ]\sin[(4k-4)\theta ]}{\cos2\theta },\\ R(1-\frac{c^{2}}{2R^{2}})\cos[(8k-7)\theta +(4k-4)\varepsilon ]), \end{array}$$

(25) $$\begin{array}{c} P_{1}^{\left(4k-2\right)}=(R(1-\frac{c^{2}}{2R^{2}})\sin[(8k-5)\theta +(4k-2)\varepsilon ],\\ 2c\cos2\theta -4c\sin\theta \frac{\cos[(4k-1)\theta ]\sin[(4k-4)\theta ]}{\cos2\theta },\\ -R(1-\frac{c^{2}}{2R^{2}})\cos[(8k-5)\theta +(4k-2)\varepsilon ]), \end{array}$$

(26) $$\begin{array}{c} P_{1}^{\left(4k-1\right)}=(-R(1-\frac{c^{2}}{2R^{2}})\sin[(8k-3)\theta +(4k-2)\varepsilon ],\\ 2c\cos2\theta +4c\sin\theta \frac{\sin(4k\theta )\cos[(4k-3)\theta ]}{\cos2\theta },\\ R(1-\frac{c^{2}}{2R^{2}})\cos[(8k-3)\theta +(4k-2)\varepsilon ]), \end{array}$$

(27) $$\begin{array}{c} P_{1}^{\left(4k\right)}=(R(1-\frac{c^{2}}{2R^{2}})\sin[(8k-1)\theta +4k\varepsilon ],\\ -2c-4c\sin\theta \frac{\sin(4k\theta )\cos[(4k-1)\theta ]}{\cos2\theta },\\ -R(1-\frac{c^{2}}{2R^{2}})\cos[(8k-1)\theta +4k\varepsilon ]). \end{array}$$

Based on the analytical ray tracing results in Equations (24)–(27), the necessary parameters in the design of DR-CMPC are determined, which are included in Section 3 below.

## 3. Parametric Design

A DR-CMPC can be uniquely defined by the parameter set of *R*, *p*, *q*, *c* and φ, where *R*, *p* and *q* are the radii of the curvature of the internal spherical surface, number of the edges and density of the *p*-sided star polygon pattern, respectively, which are the same as in the traditional CMPC; *c* determines the longitudinal distance between the two rows of reflection spots (the distance is equal to 4*c*, generally); and φ is the angle between the entrance and exit port in the x-O-z plane, which determines the effective OPL (Figure 2b). Other necessary parameters that describe the characteristics of the cell, such as incident angle *θ* and effective optical path length $L_{eff}$, can be derived from this parameter set.

### 3.1. Incident Angle θ

The incident angle $\theta_{0}$ is only determined by the *p* and *q* of the regular star polygon pattern in traditional CMPC, which can be calculated by Equation (10). However, as our calculation shows, in DR-CMPC, the incident angle *θ* also depends on parameters *R* and *c*. That means that the results in Equation (10) need to be corrected or the reflection beam will not exit the cell after the right number of passes. The correction can be expressed as:

(28) $$\theta =\kappa (R,c)•\theta_{0},$$

where κ(R,c) is the correction factor of the incident angle within the DR-CMPC, which can be derived from the analytical tracing results. We set p=4k−2(k=1,2…). After 2*p* passes, the reflection beam comes back to a point that is near the incident point. The aim of the incident angle correction is to make the two points exactly coincide with each other in spatial dimensions under the conditions of second-order approximation. One can calculate the coordinate values after 2*p* passes in traditional and double-row cases using Equations (9) and (27), respectively:

(29) $$P_{1,tra}^{\left(2p\right)}=(R\sin(4p-1)\theta_{0},0,R\cos(4p-1)\theta_{0}),$$

(30) $$\begin{array}{c} P_{1,dou}^{\left(2p\right)}=(R(1-\frac{c^{2}}{2R^{2}})\sin[(4p-1)\theta +2p\varepsilon ],-2c\cos^{2}\theta ,\\ -R(1-\frac{c^{2}}{2R^{2}})\cos[(4p-1)\theta +2p\varepsilon ]) \end{array}.$$

If we disregard the difference in coefficients and compare the x- and z-coordinate values in Equations (29) and (30), we can find that in the x-O-z plane, the point of 2*p*th reflection within the double-row cell experiences an extra small angle 2pε compared to the traditional case. Because it is clear that the reflection beam can return to the incident point after exactly 2*p* passes in the traditional cell, we need to compensate for the extra angle:

(31) $$(4p-1)\theta_{0}=(4p-1)\theta +2p\varepsilon .$$

Thus, we can obtain the analytical expression of κ(R,c) in the second-order approximation as:

(32) $$\kappa =\frac{\theta }{\theta_{0}}\approx 1-\frac{c^{2}}{R^{2}}\frac{2p\sin2\theta_{0}}{(4p-1)\theta_{0}}.$$

The correction results calculated by Equation (32) are consistent with the simulation results from the ray tracing software TracePro in the different parameter sets of *R*, *p*, *q* and *c* that we chose to verify the expression. Furthermore, the maximum deviation between the κ(R,c) from Equation (32) and the simulation results is 2.17 × 10^−4^. Therefore, the inaccuracy caused by the second-order approximation can be neglected in the parametric design.

### 3.2. Stability Analysis: Upper Limit of c

**Figure 3:**
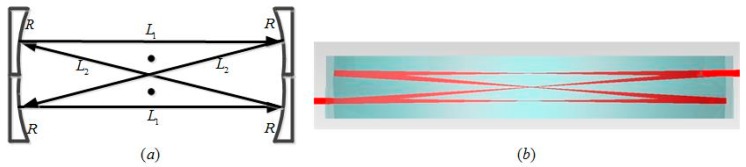
(**a**) Equivalent optical cavity and the corresponding equivalent process of the beam round trip of the DR-CMPC, where *L*_1_ is the path-length of the first and third pass in the four-pass-cycle, and *L*_2_ is the path-length of the second and forth pass in the four-pass-cycle; (**b**) The simulation example of first eight passes within DR-CMPC with *R* = 125 mm, *p* = 150, *q* = 73 and *c* = 4.4 mm, to verify the equivalent.

Parameter *c* determines the longitudinal distance between the reflection spots within DR-CMPC, which is 4*c* in the case of *c* = *c’*, which is shown in Figure 2a. Furthermore, the traditional CMPC can be seen as a particular case with *c* = 0. Parameter *c* should not be too large because the second-order approximation requires that *c << R*. Furthermore, the range of *c* is limited by the stability conditions of the DR-CMPC, which can be derived by the ABCD matrix method that is commonly used in the analysis of a laser cavity. The round trip of a beam within the DR-CMPC can be described according to the following process: a beam propagates freely over a distance of *L*_1_, before being reflected by a spherical surface with a radius of curvature of *R*. After this, the beam propagates freely over a distance of *L*_2_, before being reflected by a spherical surface with a radius of curvature of R within an equivalent optical cavity shown in Figure 3. *L*_1_ and *L*_2_ can be determined by Equations (24)–(27) in the second-order approximation as:

(33) $$L_{1}=2R(1-\frac{c^{2}}{2R^{2}})\cos\theta ,$$

(34) $$L_{2}=2R(1-\frac{c^{2}}{2R^{2}}+\frac{2c^{2}}{R^{2}}\cos2\theta )\cos\theta .$$

The transfer characteristics of a Gaussian laser beam within the equivalent optical cavity can be described by the ABCD matrix as:

(35) $$\begin{array}{l} T=\left[\begin{array}{cc} A & B\\ C & D \end{array}\right]=\left[\begin{array}{cc} 1 & 0\\ -\frac{2}{R} & 1 \end{array}\right]\left[\begin{array}{cc} 1 & L_{2}\\ 0 & 1 \end{array}\right]\left[\begin{array}{cc} 1 & 0\\ -\frac{2}{R} & 1 \end{array}\right]\left[\begin{array}{cc} 1 & L_{1}\\ 0 & 1 \end{array}\right]\\ =\left[\begin{array}{cc} 1-\frac{2L_{2}}{R} & L_{1}+L_{2}-\frac{2L_{1}L_{2}}{R}\\ -\frac{4}{R}+\frac{4L_{2}}{R^{2}} & -\frac{2}{R}(L_{1}+L_{2}-\frac{2L_{1}L_{2}}{R})+1-\frac{2L_{1}}{R} \end{array}\right] \end{array}.$$

To ensure that the beam size will not widen significantly within the cell, the element A and D of the ABCD matrix should satisfy the following condition [22]:

(36) $$-1<\left(\frac{A+D}{2}\right)<1.$$

Thus, we obtain that:

(37) $$0<(1-\frac{L_{1}}{R})(1-\frac{L_{2}}{R})<1.$$

**Figure 4:**
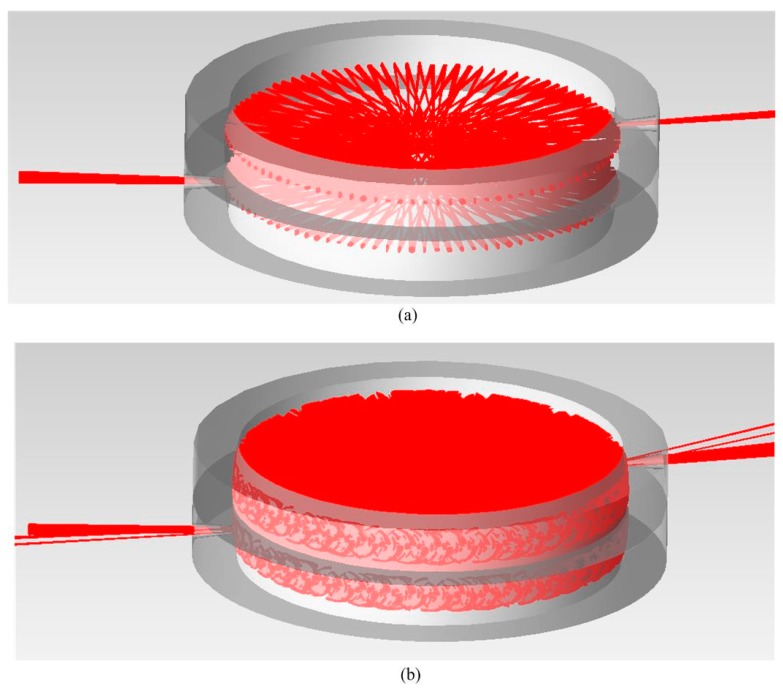
Simulation cases with parameters of *R* = 100 mm, *p* = 98 and *q* = 47 (upper limit calculated by Equation (38) is 6.477 mm) and (**a**) *c* = 6.4 mm, within the upper limit; (**b**) *c* = 6.5 mm, beyond the upper limit. The initial diameter of the beam is 6 mm, the incident focal length is 203.2 mm and the total number of rays that are traced in the simulation is 2611.

We substituted the *L*_1_ and *L*_2_ that were determined by Equations (33) and (34) into Equation (37), which allows us to the derive the stability condition of DR-CMPC in the second-order approximation as:

(38) $$c<c_{sta}=R\sqrt{\frac{2(1-\cos\theta_{0})}{\left(1-2\cos\theta_{0})(1-2\cos2\theta_{0}\right)}},$$

where *c_sta_* is the upper limit of *c*. In Figure 4, we demonstrated the simulation cases of *c* within/beyond the upper limit, while the parameter set of *R*, *p* and *q* remains unchanged. The reflection spots in the former would not spread significantly and all of the rays can pass through the exit hole after the desired pass number. The reflection spots in the latter spread massively and overlap each other, which indicates that a significant proportion of rays will not reach the correct pass number before they exit the cell.

**Figure 5:**
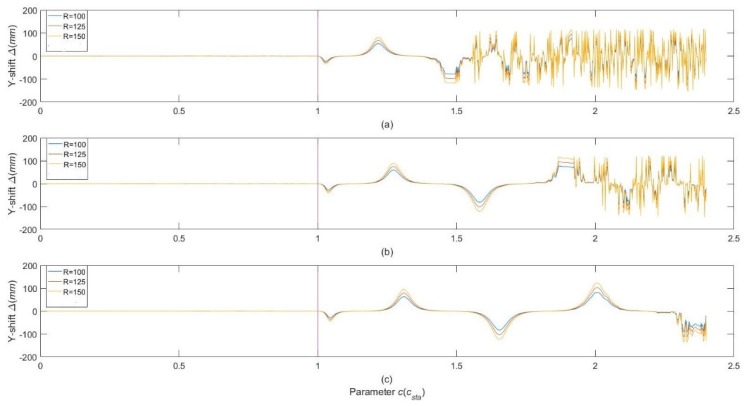
**Figure5.** Dependency of Δ upon the parameter with different parameter sets of: (**a**) *p* = 98, *q* = 47; (**b**) *p* = 150, *q* = 73; (**c**) *p* = 198, *q* = 97, where Δ is the Y-shift of chief ray exit point, *c* is the distance between the center of curvature and the plane that the reflection spots lie on, and *c_sta_* is the upper limit of *c* that calculated by Equation (38). Curves with different colors indicate different radii of curvature, while the black line indicates the boundary divided by *c_sta_*. One can see that when $c\leq c_{sta}$, the Y-shift of exit point is close to zero and varies weakly as *c* increases. When $c>c_{sta}$, the Y-shift varies significantly and frequently as *c* increases. Therefore, *c_sta_* can be regarded as the upper limit of *c* in parametric design.

Another effect caused by c exceeding the upper limit is that the y-coordinate value of the chief ray exit point would have an obvious shift from the predefined position of the exit hole, 2*c*, so that the chief ray of the reflection beam cannot exit the cell at the desired number of passes. We define the shift as:

(39) $$\Delta =y^{\left(2p-1\right)}-2c,$$

where *y*^(2*p*−1)^ is the y-coordinate value of the (2*p* − 1)th reflection point, which is usually chosen to be the exit point to achieve the maximum effective OPL. The dependency of Δ upon the parameter *c* with different parameter sets of *R*, *p* and *q* was determined by numerical ray tracing simulations, which are shown in Figure 5. One can see that the Y-shift Δ is approximately proportional to the radii of curvature *R*, especially for a relatively small *c*. Although the shape of the curves change for different *p* and *q*, *c_sta_* in Equation (38) can always be regarded as the upper limit of the range that occurs with the Y-shift Δ towards 0.

### 3.3. Projection of Angle between Incident and Exit φ

**Figure 6:**
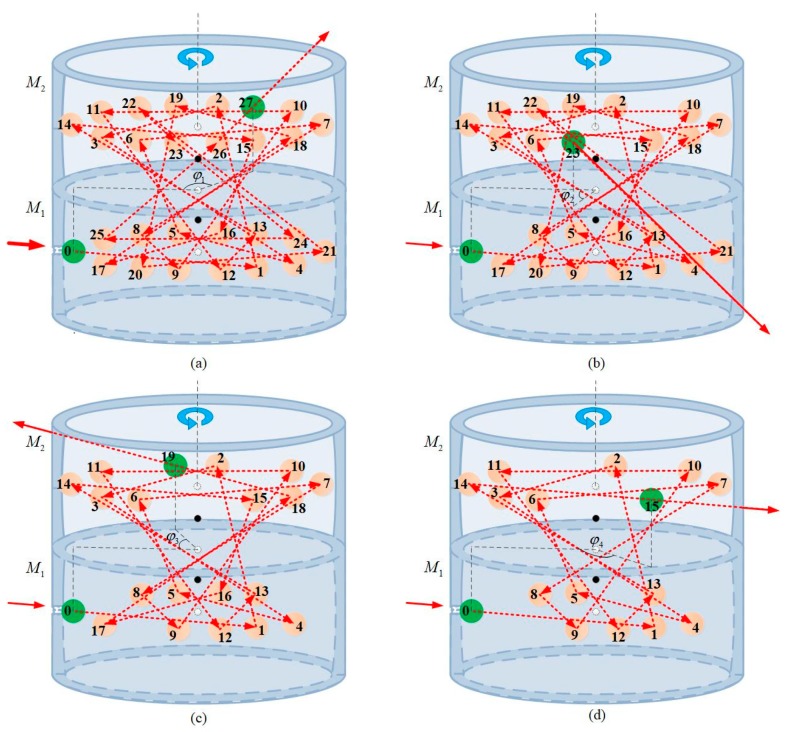
Constant interval adjustment of pass number in a cell with *p* = 14 and *q* = 5 by rotating φ around the axis of rotational symmetry (as indicated by blue rotation arrows) to change $M_{2}$ to: (**a**) $\varphi_{1}=\pi -2\theta$, corresponding to 27 passes; (**b**) $\varphi_{2}=\pi -10\theta$, corresponding to 23 passes; (**c**) $\varphi_{3}=\pi -18\theta$, corresponding to 19 passes; (**d**) $\varphi_{4}=\pi -26\theta$, corresponding to 15 passes within the cell, respectively, where φ is the projection of angel between incident and exit. As the parameter set *R*, *p*, *q* and *c* remains the same in all four cases, the constant interval adjustment of effective optical path length (OPL) is achieved in the meantime. Furthermore, the required precision of angle adjustment is *θ*, which is namely the angular interval of adjacent spots. This is approximately 25.7° in this example.

Parameter φ is the angle between the center of incident and exit holes projected into the x-O-z plane. It determines the overall number of passes and thus, the effective OPL within the DR-CMPC when the parameter set of *R*, *p*, *q* and *c* is predefined. One can change φ by rotating the $M_{2}$ around the Y-axis (examples illustrated in Figure 6) to make the exit hole overlap with pre-designed positions with a different serial number of beam passes. As a result, the beams that exit through the exit hole experience a different number of passes, which result in different effective OPL within the cell. As the serial number changes regularly with a change in φ, the constant interval adjustment of effective OPL can be achieved with the precision of rotation angle only required to be within a few or tens of degrees (namely the angular interval of the adjacent spots on the mirrors). One can set φ as:

(40) φ=π−2(1+4n)θ,(n=0,1,2,…,q),

to arrive at the corresponding overall number of passes *N* as:

(41) 2p−(1+4n),(n=0,1,2,…,q).

It is clear that when we set *n* = 0, the maximum overall number of passes (2*p* − 1) can be achieved, which is nearly twice the number achieved in the traditional CMPC (*p)*. As the beam reflects in a cycle of four within the double-row cell, the effective OPL for different number of passes can be expressed as:

(42) $$L_{eff}^{\left(N\right)}=\{\begin{array}{c} 8(k-1)R(1-\frac{c^{2}}{2R^{2}}+\frac{c^{2}}{R^{2}}\cos2\theta )\cos\theta (N=4k-4,k=1,2,\ldots )\\ \left(8k-6)(R-\frac{c^{2}}{2R})\cos\theta +8(k-1)\frac{c^{2}}{R}\cos2\theta \cos\theta (N=4k-3,k=1,2,\ldots \right)\\ \left(8k-4)R(1-\frac{c^{2}}{2R^{2}}+\frac{c^{2}}{R^{2}}\cos2\theta )\cos\theta (N=4k-2,k=1,2,\ldots \right)\\ \left(8k-2)(R-\frac{c^{2}}{2R})\cos\theta +(8k-4)\frac{c^{2}}{R}\cos2\theta \cos\theta (N=4k-1,k=1,2,\ldots \right) \end{array},$$

respectively, in second-order approximation.

## 4. Simulation

**Figure 7:**
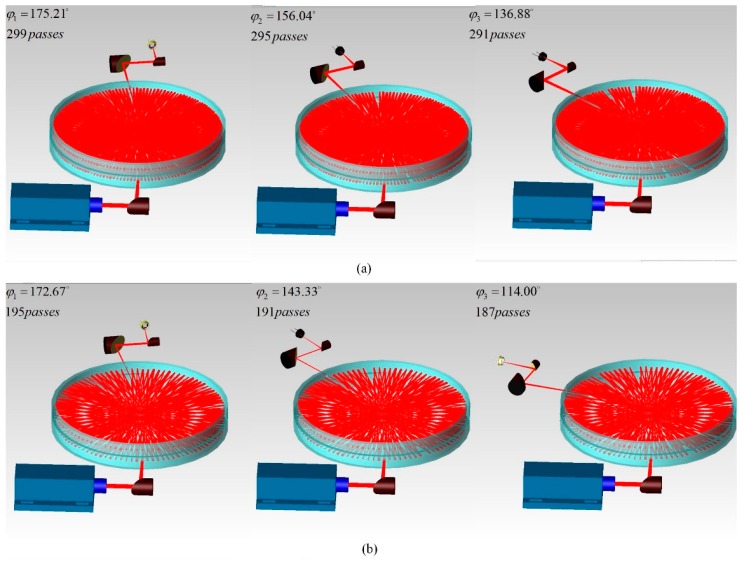

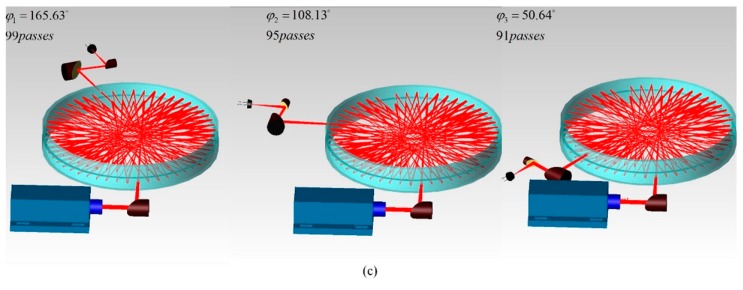
Simulations of overall optical systems based on DR-CMPC, with parameters of *R* = 125 mm, *c* = 4.4 mm and (**a**) *p* = 150, *q* = 73 and *φ*_1_ = 175.21° corresponding to 299 passes, 74.72 m effective OPL; *φ*_2_ = 156.04° corresponding to 295 passes, 73.72 m; and *φ*_3_ = 136.88° corresponding to 291 passes, 72.72 m; (**b**) *p* = 98, *q* = 47 and *φ*_1_ = 172.67° corresponding to 195 passes, 48.67 m; *φ*_2_ = 143.33° corresponding to 191 passes, 47.68 m; and *φ*_3_ = 136.88° corresponding to 187 passes, 46.68 m; (**c**) *p* = 50, *q* = 23 and *φ*_1_ = 165.63° corresponding to 99 passes, 24.57 m; *φ*_2_ = 108.13° corresponding to 95 passes, 23.57 m; and *φ*_3_ = 50.64° corresponding to 91 passes, 22.58 m. For clarity, all of the bracing/adjustment assemblies and gas circuits are not shown.

Based on the analytical tracing and parameter analysis above, we set the radius of curvature *R* = 125 mm and row interval parameter *c* = 4.4 mm, while (*p*, *q*) = (150, 73), (98, 47) and (50, 23), respectively. The simulations of overall optical systems based on DR-CMPC were run by the TracePro software, as shown in Figure 7. For better illustration, the diameter of the collimated output beam from the source was set to be 6 mm, which is much larger than the diameter commonly used in practice (usually ~2.5 mm). This means that the spot interval in practice is significantly smaller than what we used in Figure 7. After this, the collimated beam was folded through the incident hole on the bottom mirror and transferred into the double-row circular cell by a 90° off-axis parabolic mirror (effective focal length of eight inches), before being focused to the midpoint of the first pass of the beam. Next, the beam experienced a predefined number of passes within the cell, before exiting the cell through the exit hole on the upper mirror. The exit beam is re-collimated by an identical 90° off-axis parabolic mirror, before another parabolic mirror with a smaller focal length (two inches) refocuses the beam onto the photon detector. It can be clearly seen that the two rows of reflection spots are uniformly distributed on the side wall of the cell. The maximum effective OPL are 74.72 m, 48.67 m and 24.57 m, respectively, which are much larger than the state-of-the-art traditional CMPC [20]. In Figure 7, we also demonstrated regular interval adjustments by changing *φ*. The interval of adjustments was set to be four passes, which corresponds to a 1 m effective OPL. The best angular accuracy requirement is merely 2.4°. The volume of the cell is about 0.99 L and thus, the maximum PVR is 7.57 cm^−2^. This can be further promoted by reducing *c* and *R*, which are currently limited by the manufacture and coating of the mirrors. For example, if we set *R* = 80 mm, *p* = 150, *q* = 73 and *c* = 2 mm, the maximum PVR of the cell can be increased to 22.65 cm^−2^. Moreover, we calculated the astigmatism of the cell (namely, the distance between the locations of the sagittal and meridional convergence points of the last reflection) in Figure 7 using a similar method introduced in reference [23]. In the 299-passes case, the astigmatism of the cell is only 9.30 × 10^−6^ mm, which is much smaller than the astigmatism of the traditional circular cell with the same *R*, *p* and *q* parameters, which is 2.08 × 10^−2^ mm.

## 5. Conclusions

The concept of DR-CMPC was proposed for the first time, which consists of two identical high-reflectance coated mirrors with spherical inner surfaces. The two mirrors are piled up vertically and share the same axis of rotational symmetry. Relevant analytical chief ray tracing analysis, stability analysis and the method for parameter determination are also presented in detail. The effective OPL of DR-CMPC is twice that of the traditional CMPC with the same diameter and interval of spots, which means that both volume and interference fringes do not increase. Furthermore, the effective OPL can be readily adjusted with regular intervals by rotating the upper mirror with an angular precision of few or tens of degrees, which avoids the rigor incident angle adjustment that requires a precision of approximately 0.01°. A spatial separation of pre- and post-transfer optical systems was achieved, especially for the maximum effective OPL case, and the overlap of optical components in a large number of passes is avoided. The designs with 74.72 m, 48.67 m and 24.57 m effective OPL are presented and verified by ray tracing simulations within a 25 cm diameter double-row cell, while the examples for regular adjustment with a 1 m interval are also provided. The overall astigmatism of the 74.72 m OPL design is only 9.30 × 10^−6^ mm, which is four orders of magnitude smaller than that in the traditional CMPC with the same *R*, *p* and *q* parameters (2.08 × 10^−2^ mm). Moreover, the stability condition of DR-CMPC is also derived by the ABCD matrix method. Therefore, the stable propagation of beams without significant dispersion in geometry can be achieved with simple spherical reflection surfaces rather than more complex reflection surfaces, such as hypertorus circular surfaces and paraboloids. Consequently, the complexity and cost of mirror fabrication are reduced. In conclusion, the relatively long effective OPL, easy adjustment, stable beam transmission and low cost of DR-CMPC suggest that it potentially has versatile applications, such as absorption spectroscopy detection, gas/liquid sensing and optical delay.
